# Mimicking Darier Disease In Vitro: A Human Epidermal Organoid Approach

**DOI:** 10.1111/exd.70191

**Published:** 2025-12-29

**Authors:** Rishika Agarwal, Erika Parente, Simon M. Müller, Elisabeth A. Kappos, Tanja Dittmar, Michael Kunz, Roni P. Dodiuk‐Gad, Nisim Asayag, Johann E. Gudjonsson, Beda Mühleisen, Emmanuel Contassot, Alexander A. Navarini

**Affiliations:** ^1^ Department of Biomedicine University Hospital and University of Basel Basel Switzerland; ^2^ Dermatology Department University Hospital of Basel Basel Switzerland; ^3^ Department of Plastic, Reconstructive, Aesthetic, and Hand Surgery University Hospital Basel Basel Switzerland; ^4^ Bruce Rappaport Faculty of Medicine Technion—Institute of Technology Haifa Israel; ^5^ Dermatology Department Emek Medical Center Afula Israel; ^6^ Division of Dermatology, Department of Medicine University of Toronto Toronto Ontario Canada; ^7^ Department of Internal Medicine University of Michigan Ann Arbor Michigan USA; ^8^ Department of Dermatology University of Michigan Ann Arbor Michigan USA; ^9^ Taubman Medical Research Institute University of Michigan Ann Arbor Michigan USA

**Keywords:** acantholysis, Darier disease, dyskeratosis, organoids

## Abstract

Darier disease (DD) is a rare genetic disorder caused by mutations in the *ATP2A2* gene, resulting in calcium dysregulation and impaired keratinocyte adhesion. Due to the paucity of suitable models, understanding the molecular mechanisms of DD has been challenging. In this study, we developed a human epidermal organoid model derived from DD patient keratinocytes to investigate the molecular and phenotypic features of the disease. The model recapitulates key aspects of DD pathology, including acantholysis, desmosomal dysfunction and barrier disruption, with mislocalisation of desmosomal proteins. Furthermore, the transcriptomic landscape of DD organoids reflected broad perturbations in epidermal structure. Enrichment of pathways associated with epidermal development, cell adhesion, cell migration and keratinocyte differentiation underscored the multifaceted disruption of epithelial integrity and homeostasis that defines DD pathology. Our work demonstrates that epidermal organoids derived from patients with Darier disease are a valuable model for studying DD. They provide a platform to study complex genetic epidermal disorders and personalised drug screening.

## 1 Background

Darier disease (DD) is a rare autosomal dominant genodermatosis characterised by loss of keratinocyte adhesion referred to as acantholysis and abnormal keratinisation (dyskeratosis). Clinically, DD presents with hyperkeratotic papules primarily affecting seborrheic areas such as the chest, back, scalp and forehead as well as specific changes of the oral and genital mucosa [1]. Extracutaneous features including ocular [2], neuropsychiatric [3] and parotid gland abnormalities [4] may be associated. Histologically, the skin of DD patients shows suprabasal acantholysis and dyskeratosis with formation of characteristic *corps ronds* and *grains*. The disease is caused by mutations in the *ATP2A2* gene encoding sarco/endoplasmic reticulum Ca^2+^‐ATPase type 2 (SERCA2), an essential pump that regulates intracellular calcium homeostasis [5]. SERCA2 dysfunction disrupts calcium signalling within keratinocytes, impairing cell–cell adhesion and desmosome assembly, which are critical for epidermal integrity.

Current treatments are largely symptomatic and include topical retinoids, corticosteroids and systemic retinoids for severe cases [6]. However, therapeutic options remain limited and no curative treatment exists. Advances in disease modelling are needed to better understand the underlying mechanisms. Mouse models have proven to be inadequate as they do not recapitulate the phenotype of the disease [7]. In this study, we aimed to fill this gap by using human epidermal organoids (HEOs) [8] derived from keratinocytes isolated from DD patients. Although our epidermal organoid model isolates keratinocyte‐intrinsic processes, it is noteworthy that several recent transcriptomic studies of Darier disease skin have reported upregulation of inflammatory and immune pathways, including Th17/IL‐23–related signalling and innate defence responses [9, 10]. These findings highlight that, in patients, keratinocyte defects coexist with secondary inflammatory changes that may amplify epidermal stress and barrier dysfunction. Our keratinocyte‐only system therefore provides a complementary approach, allowing the dissection of primary desmosomal and differentiation abnormalities independent of immune contributions, which could be further explored in future combined models.

## 2 Questions Addressed

We aimed to establish and characterise a HEO model derived from keratinocytes of DD patients. Specifically, we sought to determine whether this model recapitulates key pathological features of DD, such as desmosomal dysfunction, acantholysis and barrier disruption. Furthermore, we aimed to investigate transcriptomic alterations associated with DD and identify dysregulated pathways that contribute to epidermal disorganisation and impaired keratinocyte function.

## 3 Experimental Design

### 3.1 Keratinocyte Isolation & Expansion

Primary keratinocytes were isolated from healthy donor (*n* = 5) or DD patient skin (*n* = 3). After dermis and subcutaneous tissue removal, the epidermis was treated with 50 U/mL Dispase (Corning, Bedford, MA) and 10% antibiotic‐antimycotic (Gibco, Thermo Fisher Scientific Inc., Waltham, MA) overnight at 4°C. The epidermal tissue was minced and incubated at 37°C in TrypLE Express (Gibco). Keratinocyte suspensions were washed twice with KSFM (EGF 5 ng/mL; BPE 25 μg/mL, Thermo Fisher Scientific Inc.), resuspended in KSFM with 10 μM Y‐27632 (Lucerna Chem, Lucern, Switzerland) and seeded in T‐25 Primaria flasks (Corning). The medium was changed every 2–3 days.

### 3.2 HEO Culture

HEOs were generated as previously described. Briefly, keratinocytes were mixed with cold basement membrane extract (BME2, Cultrex, R&D Systems, Biotechne, Minneapolis, MN) in a ratio of 1:3 and plated at 3000 cells/10 μL in low‐adhesion 24‐well plates (Greiner Bio‐One, Kremsmünster, Austria). HEO medium included advanced DMEM/F12, Antibiotic‐Antimycotic, Hepes, GlutaMAX, B27 supplement (Gibco), N‐Acetylcysteine‐1 (1 mM, SigmaAldrich, St. Louis, MO, USA), Noggin (50 ng/mL, Qkine, Cambridge, UK), R‐spondin 1 (50 ng/mL, Qkine), FGF10 (100 ng/mL, Qkine), Heparin (0.0001%, Stemcell Technologies, Vancouver, BC, Canada), Forskolin (10 nM, Tocris, Biotechne), Rho kinase inhibitor Y‐27632 (10 μM, Lucerna Chem), Primocin (50 mg/mL, Invivogen, San Diego, CA). The HEOs were cultured for 7 days.

### 3.3 Immunofluorescence

HEOs were retrieved from BME2 with 1.5 U/mL Dispase II (Sigma Aldrich), fixed in 4% PFA and incubated in cold PBS + 0.1% Tween 20 for 10 min. Samples were blocked in blocking buffer (0.001% Triton X‐100/0.2% BSA) for 15 min. Primary and secondary antibodies (Table S1) were applied sequentially; nuclei were counterstained with DAPI. Organoids were mounted in 60% glycerol and 2.5 M fructose solution using a 0.3 mm iSpacer (SunjinLab, Taiwan).

### 3.4 Microscopy and Image Analysis

Brightfield images were captured using a Nikon Ti2 widefield microscope (4×, 20×, 40×; DS‐Ri2, RGB CMOS camera) and NIS AR software. Immunofluorescence confocal images were acquired with the Nikon X‐light V3 spinning disc microscope. Software used for image analysis also includes FIJI281 and Imaris (RRID:SCR_007370).

### 3.5 RNA Isolation

BME2 droplets were dissolved in TrypLE Express (Thermo Fisher Scientific Inc.). Organoids were then collected and washed with PBS twice and resuspended in RLT buffer (Qiagen, Hilden, Germany) with 1% β‐mercaptoethanol (Sigma Aldrich). Total RNA was extracted from organoids using an RNeasy Mini Kit (Qiagen, Hilden, Germany) and from skin using an RNeasy fibrous tissue Mini Kit (Qiagen) according to the manufacturer's protocol.

### 3.6 Western Blot

Proteins were isolated with RIPA buffer plus protease inhibitors, mixed with Lämmli buffer and resolved by SDS‐PAGE. Thirty micrograms of protein per slot were loaded and transferred to membranes by wet blotting, which were then blocked in 5% milk in TBS‐T, incubated with primary antibody (to SERCA2 and GAPDH, Table S1) overnight at 4°C, then with an HRP‐conjugated secondary antibody (Table S1) for 2 h at room temperature. Detection was via chemiluminescence (SuperSignal West Pico Plus, ThermoScientific) and analysed with FUSION FX7 EDGE machine and software (Witec) with GAPDH as a control.

### 3.7 Biotin Penetration Assay

HEOs were treated with 50 U/mL Dispase, incubated at 37°C, washed and exposed to 1 mg/mL EZ‐link Sulfo‐NHS‐biotin in PBS with 1 mM CaCl_2_ for 1 h. After fixation (4% PFA), HEOs were permeabilised with 0.1% Tween‐20, blocked and stained overnight at 4°C with occludin antibody (Cell Signalling). Secondary antibody and Alexa Fluor 647‐Streptavidin staining followed. HEOs were mounted, and the images were acquired as described above and biotin penetration was quantified as detailed in the Supporting Information section.

### 3.8 RNA Sequencing

mRNA was purified from total RNA using poly‐T beads. cDNA was synthesised with random hexamer primers; directional or non‐directional libraries were prepared through standard workflows (end repair, A‐tailing, adapter ligation, size selection, amplification). Libraries were validated (Qubit, qPCR, bioanalyzer), pooled and sequenced on Illumina platforms. Data were aligned to reference genomes using Hisat2, with gene counts by featureCounts and FPKM values calculated. Data analyses are described in Supporting Information.

### 3.9 Statistics

**FIGURE 1:**
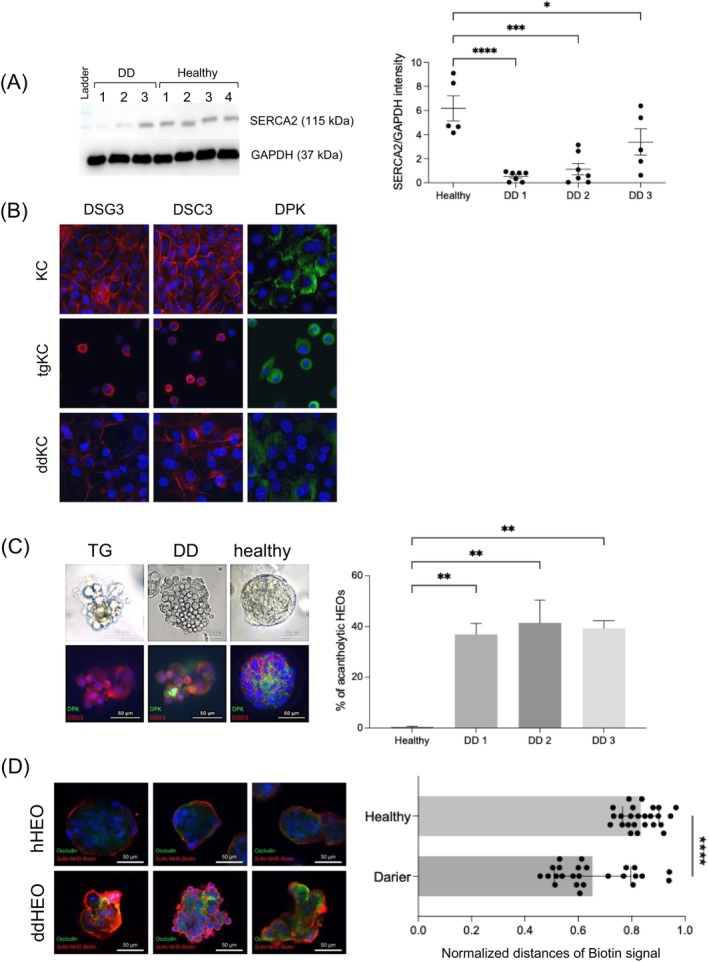
Defective desmosome expression, acantholysis and permeability in DD patient‐derived HEOs. (A) Reduced SERCA2 expression in 2D cultures of keratinocytes isolated from three patients suffering from DD (P1, P2 and P3). One representative blot is shown (left panel). SERCA2 band intensity/GAPDH was measured in several 2D cultures of healthy keratinocytes (*n* = 5) and several 2D cultures of DD keratinocytes from three patients (P1, *n* = 7; P2, *n* = 7; P3, *n* = 5). (B) Immunofluorescence of the desmosome proteins DSC3, DSG3 and DPK in 2D cultures of healthy (KC), TG‐treated (tgKC) and DD keratinocytes (ddKC). Representative images of four independent 2D cultures of each type. Blue = DAPI. (C) Brightfield images of acantholytic‐like HEOs treated with TG and generated from a DD patient. Acantholytic and healthy‐looking HEOs were numerated in three independent 3D cultures of the three patient‐derived HEOs. HEOs from healthy skin donors were unaffected (mean ± SD of three healthy donors is shown). Fifty HEOs per culture were counted. Below, immunofluorescence of DSG3 and DPK revealed a cytoplasmic localisation in TG‐treated HEOs and DD‐HEOs whereas, DSG3 and DPK were localised at the surface and between cells as expected in healthy HEOs non treated with TG. (D) Biotin‐penetration assay showed that in contrast to healthy HEOs, DD‐HEOs were not able to keep biotin at the periphery but were instead highly permeable to it. Permeability was quantified by measuring the distance of the biotin signal to the centre of the HEOs. *p < 0.05, **p < 0.01, ***p < 0.001, ****p < 0.0001.

Comparison of SERCA2 band intensity (Figure 1A), percentage of acantholytic HEOs (Figure 1B) and biotin penetration (Figure 1C) has been performed using one‐way ANOVA with post hoc Tukey using GraphPad Prism.

## 4 Results

We collected fresh skin biopsies from lesional areas of three patients with Darier disease (DD) and isolated primary keratinocytes. SERCA2 expression was assessed in early passage (passage 2–3) DD keratinocytes using Western blotting (Figure 1A). Keratinocytes derived from five healthy donors (HD) served as controls. SERCA2 expression was markedly reduced in DD keratinocytes compared to HD controls, although non‐negligible inter‐donor variability was observed among the DD samples. To investigate the impact of SERCA2 dysfunction on desmosomal integrity, we analysed the localisation of desmosomal proteins desmoglein 3 (DSG3), desmocollin 3 (DSC3) and desmoplakin (DPK) in 2D cultures of keratinocytes from HD treated or not with TG, and DD patients using confocal microscopy (Figure 1B). In HD keratinocytes, all three desmosomal proteins localised strongly to the plasma membrane, consistent with their role in mediating cell–cell adhesion. In contrast, keratinocytes derived from DD patients showed reduced and irregular membrane expression of DSG3 and DSC3, with significant cytoplasmic retention. DPK was predominantly mislocalised to the cytoplasm, indicating impaired desmosome assembly or trafficking as previously suggested [11].

To assess whether these abnormalities extended to three‐dimensional cultures, we examined desmosomal protein localisation in HEOs derived from DD keratinocytes and in Thapsigargin (TG)‐treated HEOs (Figure 1C). 40% of the HEOs generated from keratinocytes isolated from DD patients had an acantholytic morphology (Figure 1C). While HD‐HEOs showed robust junctional expression of DSG3, DSC3 and DPK throughout the stratified layers, DD‐HEOs displayed patchy, cytoplasmic staining of all three proteins, particularly in suprabasal layers. Similarly, TG treatment of HD‐HEOs led to a comparable mislocalisation pattern, in line with the causative role for SERCA2 inhibition in desmosomal disorganisation [12]. Considering that epidermal barrier dysfunction is a hallmark of DD skin [13], we assessed the barrier properties of DD‐HEOs using a biotin permeability assay [14]. In HD‐HEOs, biotin was restricted to the stratum basale and the periphery. In contrast, in a substantial proportion of DD‐HEOs, biotin was able to penetrate throughout the entire 3D structure, including the stratum corneum, indicating a compromised barrier (Figure 1D).

Collectively, these observations show that HEOs derived from DD patient keratinocytes recapitulate key pathological features of DD in vitro, including acantholysis, desmosome protein mislocalisation and barrier disruption.

**FIGURE 2:**
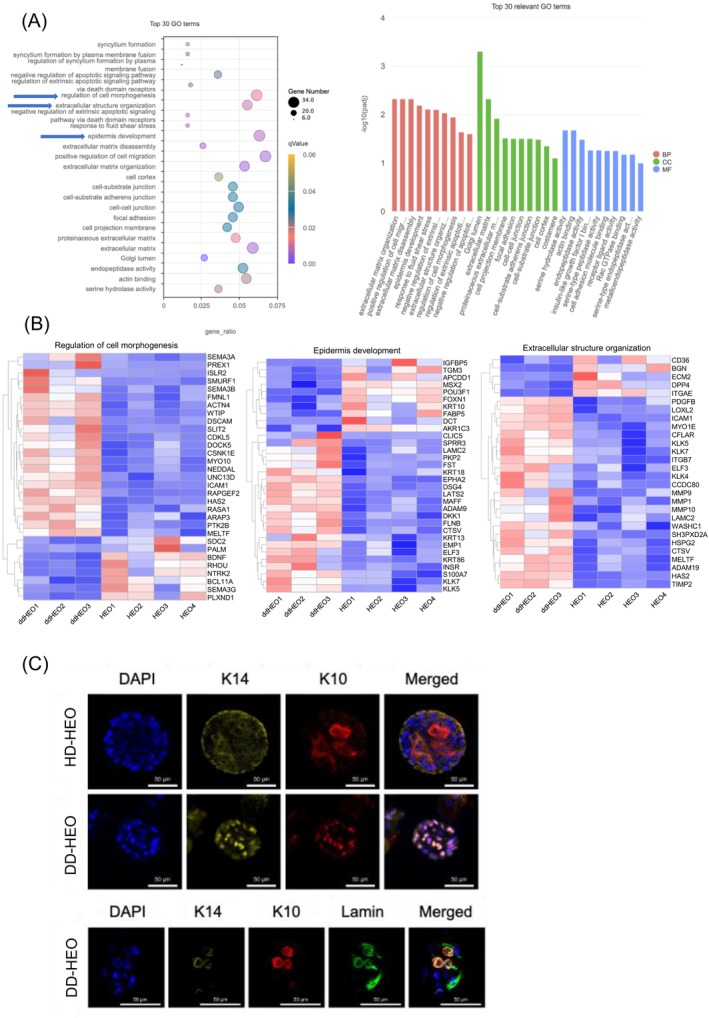
RNAseq and keratin expression revealed major abnormalities in epidermal development and structural integrity in DD HEOs. (A) GO enrichment analysis of DEGs in DD HEOs versus Healthy HEOs. Highest gene ratios were related to regulation of morphogenesis, epidermis development and extracellular structure. (B) Expression heatmaps of the DEGs involved in *regulation of morphogenesis*, *epidermis development* and *extracellular structure*. (C) Immunofluorescence show that DD‐HEOs have mislocalised keratin 10 (red) and 14 (yellow). They are co‐expressed in the nuclei (DAPI, blue) and the perinuclear space as confirmed by co‐staining with lamin (green), a nuclear membrane marker. Representative images from three DD patients.

To further characterise epidermal organoids derived from DD patients, we performed RNA sequencing on the three DD patients and compared their transcriptomic profiles with those of healthy donors (HD, *n* = 5). RNA sequencing analysis comparing DD epidermal organoids with healthy controls revealed significant enrichment of gene ontology (GO) categories associated with key biological processes. Notably, the terms *regulation of cell morphogenesis, epidermis development, positive regulation of cell migration* and *extracellular matrix organisation* emerged as among the most significantly altered pathways (Figure 2). The GO term *regulation of cell morphogenesis* was significantly upregulated in DD organoids. This finding is consistent with the well‐established histomorphologic alterations of keratinocytes in DD showing a rounded shape, dyskeratoses with formation of corps ronds and grains and suprabasal acantholysis, reflecting an underlying defect in the ability of cells to maintain and regulate their morphogenetic programme. The resulting dyskeratosis, including the formation of corps ronds and grains, reflects an underlying defect in the ability of cells to maintain and regulate their morphogenetic programme. Disruption of desmosomal adhesion complexes, a hallmark of DD, likely contributes to these changes in cell shape and structural cohesion [15, 16]. The enrichment of the *epidermis development* category in the RNAseq dataset further supports the idea that DD involves a profound impairment in epidermal differentiation. In line with abnormal epidermal development, the expression of the keratin‐10 (*K10*) gene is significantly reduced in DD‐HEOs (Figure 2B). Moreover, K10 as well as K14 display an atypical nuclear/perinuclear localisation in DD‐HEOs, rather than the expected cytoplasmic distribution observed in HD‐HEOs (Figure 2C). The orderly progression of keratinocytes from the basal layer to the stratum corneum is therefore disrupted, leading to architectural disarray and an incomplete/dysfunctional epidermal barrier. These defects not only account for the compromised structural integrity of the epidermis in DD but also help explain the skin's susceptibility to infections and environmental insults [6]. The GO term *positive regulation of cell migration* was also significantly represented in DD organoids, suggesting an upregulation of signalling pathways that promote cell motility as a possible attempt to repair the affected epidermis. Finally, a significant enrichment of genes involved in *extracellular matrix organisation* suggests that the extracellular matrix milieu is actively remodelled in DD organoids. Although the disease primarily affects cell–cell adhesion, repeated cycles of acantholysis and epidermal injury likely activate tissue repair responses involving matrix turnover. Matrix metalloproteinases (MMPs), particularly MMP‐9, have been detected in DD lesions and are known to be involved in the degradation and remodelling of basement membrane and intercellular matrix components [17]. Altered expression of ECM‐related genes in our data set may reflect the interplay between keratinocyte dysfunction and tissue remodelling in DD. These observations are in alignment with known aspects of DD pathophysiology and may provide insight into the broader cellular programmes disrupted in diseased epidermis.

**FIGURE 3:**
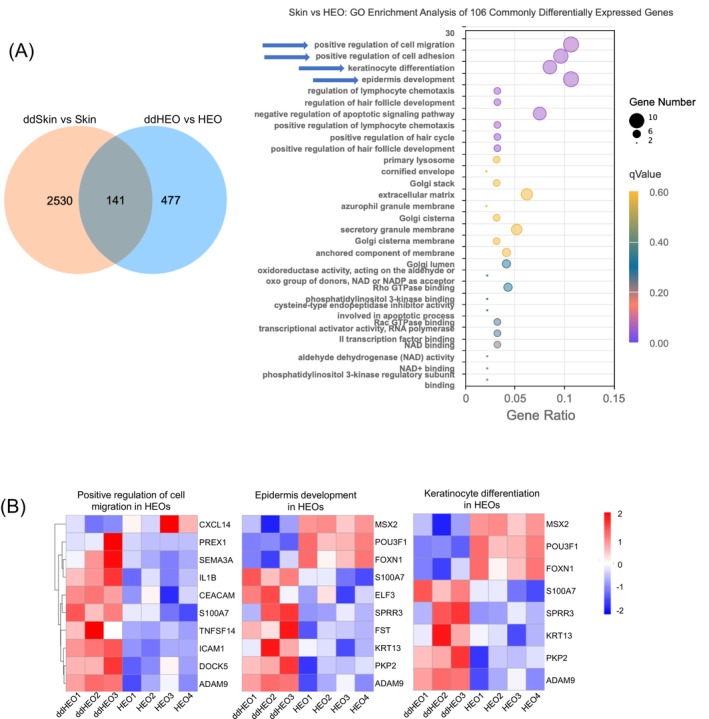
Common DEGs in DD‐HEOs and DD‐skin vs their healthy counterparts are related to skin development and structural integrity. (A) Left panel: Venn diagram showing unique and common DEGs between DD skin over healthy and DD HEOs vs healthy HEOs. Among the 141 common DEGs, 106 were found to be similarly regulated, either up or down. Right panel: GO enrichment analysis of the 106 common genes regulated in the same way, either up or down. Highest gene ratios were related to regulation of positive regulation of cell migration, positive regulation of cell adhesion, and keratinocyte differentiation. (B) Expression heatmaps of the DEGs involved in regulation of positive regulation of cell migration, positive regulation of cell adhesion, and keratinocyte differentiation.

Then, we compared the differentially expressed genes in DD‐HEOs to the skin of DD patients. There were 141 differentially expressed genes in common between DD HEOs and DD skin (Figure 3), of which 106 were regulated in the same way, either up or down. GO analysis of these common genes revealed the enrichment of the terms *epidermis development*, *positive regulation of cell migration, positive regulation of cell adhesion and keratinocyte differentiation*. Again, these biological processes closely align with the established hallmarks of DD pathogenesis and reflect the transcriptomic signatures of keratinocyte dysfunction and chronic skin damage observed in the disease. While DD is best known for defective cell adhesion due to impaired desmosomal function and resulting suprabasal acantholysis, the upregulation of pro‐adhesion pathways may represent a compensatory response to this defect as suggested by the *positive regulation of cell adhesion* enrichment. The enrichment of genes involved in *keratinocyte differentiation* is consistent with the premature and aberrant differentiation processes known to occur in DD [18]. Indeed, the disease disrupts normal keratinocyte maturation pathways, which are crucial for building an effective epidermal barrier. Altogether, transcriptomic profiling of DD epidermal organoids reveals upregulation of pathways closely associated with key aspects of DD pathogenesis, including impaired morphogenesis, disordered differentiation, chronic inflammation and extracellular matrix remodelling.

## 5 Conclusion and Perspectives

Our DD HEO model replicates key pathological features of Darier disease, including acantholysis, desmosomal mislocalisation, barrier dysfunction and transcriptomic dysregulation. It offers a human‐relevant platform to study DD's pathogenesis and serves as a promising tool for high‐throughput drug screening and personalised therapeutic development.

## Author Contributions

R.A. and E.P. performed the experiments. R.A., E.P., E.C. and B.M. analysed the data. T.D. assisted with the experiments. E.A.K., S.M.M., M.K. and N.A. collected the samples. R.P.D.‐G. and J.E.G. collected samples and reviewed the manuscript. A.A.N. and E.C. designed the experiments, analysed the data and wrote the manuscript.

## Ethics Statement

The study was approved by the Ethics Committee (Ethikkommission Nordwest‐ und Zentralschweiz, approval number 2021‐01616). All procedures were in accordance with the Declaration of Helsinki principles.

## Consent

All patients and healthy donors gave their written informed consent.

## Conflicts of Interest

A.A.N. declares that, in the last 3 years, he has been a consultant and advisor and/or received speaking fees and/or grants and/or served as an investigator in clinical trials for AbbVie, Almirall, Amgen, Biomed, BMS, Boehringer Ingelheim, Canfield, Eli Lilly, Galderma, Incyte Biosciences, Janssen‐Cilag, LEO Pharma, Louis Widmer, Merz Pharma, MSD, Novartis, Pfizer, Pierre Fabre Pharma, Sanofi, Takeda and UCB. S.M.M. declares that in the last 3 years, he has been a consultant and advisor and/or received speaking fees and/or grants and/or served as an investigator in clinical trials for Sanofi‐Aventis AG, Galderma SA, Janssen‐Cilag AG, LEO Pharmaceutical Products, Amgen, Incyte, Novartis Pharma AG, Pfizer and UCB. B.M. declares that in the last 3 years, he has been a consultant and advisor and/or received speaking fees and/or grants and/or served as an investigator in clinical trials for Galderma SA, LEO Pharmaceutical Products, Pierre Fabre Pharma and Novartis Pharma AG.

## Supporting information


**TABLE S1:** Antibodies used in the study.

**Data S1:** exd70191‐sup‐0002‐Supinfo.docx.

## Data Availability

The data that support his study is available from the corresponding author upon reasonable request.
