# Supplementary material for: Mimicking Darier Disease In Vitro: A Human Epidermal Organoid Approach
Source: Exp Dermatol. 2025 Dec 29;34(12):e70191. doi: 10.1111/exd.70191 (PMC12749551; doi:10.1111/exd.70191)
Supplement: Supplementary file 1 — TABLE S1: Antibodies used in the study. [file EXD-34-e70191-s001.docx]

|  | **Application** | **Vendor** | **Clone** |
| --- | --- | --- | --- |
| Desmoplakin | IF | Abcam | EPR4383(2) |
| Desmoglein 3 | IF | Abcam | 3G133 |
| Desmocollin 3 | IF | Progen | Dsc3-U114 |
| Occludin | IF | Cell Signaling Technology | E6B4R |
| SERCA2 | WB | Abcam | EPR9392 |
| GAPDH | WB | Abcam | EPR16891 |
| **Secondary antibodies** |  | **Vendor** | **Clone** |
| Donkey Anti-Rabbit AF 488 | IF | Jackson Immuno Research | Polyclonal |
| Goat Anti-Guineapig AF 568 | IF | Abcam | Polyclonal |
| Donkey Anti-Mouse AF 647 | IF | Jackson Immuno Research | Polyclonal |
| Goat Anti-Rabbit-HRP | WB | Abcam | Polyclonal |

Table S1: Antibodies used in the study
