# Supplementary material for: Mimicking Darier Disease In Vitro: A Human Epidermal Organoid Approach
Source: Exp Dermatol. 2025 Dec 29;34(12):e70191. doi: 10.1111/exd.70191 (PMC12749551; doi:10.1111/exd.70191)
Supplement: Supplementary file 2 — Data S1: exd70191‐sup‐0002‐Supinfo.docx. [file EXD-34-e70191-s002.docx]

**Supplementary methods**

Biotin penetration analysis

The analysis was conducted using images acquired with a confocal microscope at 20× magnification. Using Fiji (ImageJ), the selected image stack was narrowed down to a representative, round organoid. This z-slice was duplicated, and the red channel was isolated and converted to 8-bit format. A circle was fitted around the organoid using the “Fit Circle” tool, saved as a Region of Interest (ROI), and used to crop the image for targeted analysis. A centered line was drawn across the organoid to define the diameter, and both the circle and line were measured to extract key parameters such as the center coordinates and diameter length. A threshold was then applied to the red signalto segment relevant structures. Particle analysis was performed to quantify the red signal. Coordinates from this analysis were exported to Excel, where distances from each point to the circle center were calculated and normalized by radius. The mean normalized distance was then computed for each organoid. Final data visualization and statistical comparisons were performed in GraphPad Prism using an unpaired, non-parametric Mann-Whitney test.

RNAseq

Poly(A)+ RNA was enriched from total RNA using oligo(dT) magnetic beads (poly-T capture) (Illumina TruSeq Stranded). Briefly, total RNA was hybridized to oligo(dT)25-functionalized magnetic beads, washed to remove rRNA and non-polyadenylated species, and heat-eluted prior to fragmentation and cDNA synthesis. Input RNA integrity was assessed by capillary electrophoresis (Agilent Bioanalyzer RNA 6000 Nano). Inclusion criteria required RNA integrity ≥5.0, with flat baseline (Agilent 2100). Libraries were sequenced on an Illumina platform with paired-end reads (150 bp) targeting ≥20 million read pairs per sample.

Differential gene expression (DEG) analysis was performed by selecting genes with an adjusted p-value (padj) below 0.05, excluding genes located on the Y chromosome. The analysis, including the intersection across multiple datasets, was conducted in Python using the pandas and numpy libraries. Visualization of DEGs through volcano plots was done in R (version 4.3.1) with the ggplot2 package, while heatmaps were generated using the pheatmap package. Pathway visualization through KEGG maps was accomplished using the pathview library in R. Chord plots were created in Python within JupyterLab (version 4.2.3), utilizing the holoviews library for image rendering and the bokeh library for legend generation.

Further visualizations—including bubble plots for GO, Reactome, KEGG, and DisGeNET enrichment analyses; Venn diagrams; principal component analysis (PCA); and correlation matrices—were produced using the NovoMagic platform (Novogene Co. Ltd, 2014–2017). To ensure reproducibility and validation, enrichment analysis was independently repeated using the g:Profiler web tool (<https://biit.cs.ut.ee/gprofiler/gost>).
